# The distribution of the therapeutic monoclonal antibodies cetuximab and trastuzumab within solid tumors

**DOI:** 10.1186/1471-2407-10-255

**Published:** 2010-06-03

**Authors:** Carol M Lee, Ian F Tannock

**Affiliations:** 1 Divisions of Applied Molecular Oncology and Medical Oncology and Hematology Princess Margaret Hospital and University of Toronto, Toronto, ON, Canada

## Abstract

**Background:**

Poor distribution of some anticancer drugs in solid tumors may limit their anti-tumor activity.

**Methods:**

Here we used immunohistochemistry to quantify the distribution of the therapeutic monoclonal antibodies cetuximab and trastuzumab in relation to blood vessels and to regions of hypoxia in human tumor xenografts. The antibodies were injected into mice implanted with human epidermoid carcinoma A431 or human breast carcinoma MDA-MB-231 transfected with *ERBB2 *(231-H2N) that express high levels of ErbB1 and ErbB2 respectively, or wild-type MDA-MB-231, which expresses intermediate levels of ErbB1 and low levels of ErbB2.

**Results:**

The distribution of cetuximab in A431 xenografts and trastuzumab in 231-H2N xenografts was time and dose dependent. At early intervals after injection of 1 mg cetuximab into A431 xenografts, the concentration of cetuximab decreased with increasing distance from blood vessels, but became more uniformly distributed at later times; there remained however limited distribution and binding in hypoxic regions of tumors. Injection of lower doses of cetuximab led to heterogeneous distributions. Similar results were observed with trastuzumab in 231-H2N xenografts. In MDA-MB-231 xenografts, which express lower levels of ErbB1, homogeneity of distribution of cetuximab was achieved more rapidly.

**Conclusions:**

Cetuximab and trastuzumab distribute slowly, but at higher doses achieve a relatively uniform distribution after about 24 hours, most likely due to their long half-lives in the circulation. There remains poor distribution within hypoxic regions of tumors.

## Background

The ErbB family of receptor kinases is a group of four trans-membrane proteins (ErbB1 - ErbB4) that share similarities in structure and are involved in signaling pathways that stimulate cellular proliferation [1]. Ligand binding induces receptor homo- and hetero-dimerization, although no ligand has been identified for ErbB2. Dimerization of the receptors stimulates their intrinsic tyrosine kinase activity resulting in receptor autophosphorylation [2]. These phosphorylated residues serve as binding sites for molecules involved in the regulation of intracellular signaling cascades. Overexpression of ErbB receptors may occur in a wide range of epithelial cancers, including those of the breast [3], colon [4], head and neck [5], kidney [6], lung [7,8], pancreas [9], prostate [10] and esophagus [11,12] and has been associated with an aggressive phenotype.

Molecular targeted agents that interact with receptor tyrosine kinases on tumor cells are used increasingly in clinical oncology. There are two classes of agents, monoclonal antibodies and low-molecular-weight tyrosine kinase inhibitors. Cetuximab (chimeric mouse/human) and trastuzumab (humanized) are monoclonal antibodies that target the extracellular domain of the receptors ErbB1 [13-16] and ErbB2 [15,17] respectively. Binding of cetuximab and trastuzumab to ErbB1 and ErbB2 respectively prevents receptor phosphorylation and activation of the kinase domain, thereby inhibiting cell proliferation [18-20]. Binding of trastuzumab to its receptor also reduces shedding of the extracellular domain of ErbB2 and prevents the production of an active truncated fragment [20-22]. These agents have shown therapeutic activity against colorectal cancer and breast cancer respectively and are in wide clinical use [21,22].

Limited penetration of drugs through tumor tissue is an important and rather neglected cause of clinical resistance to chemotherapy [23-25]. Drug distribution from blood vessels within tumors depends on diffusion and and/or convection, and is inhibited by consumption in proximal cells [23,25-27]; for monoclonal antibodies consumption is due to binding to the receptor target, which is dependent on antibody dose, number of antigenic targets per cell, and the affinity of the antibody for its target [28]. Convection depends on gradients of pressure (both hydrostatic and osmotic) between the vascular space and the interstitial space, while diffusion depends on molecular size, shape and concentration gradients [26,27]. Because monoclonal antibodies are large molecules they might be expected to have poor distribution from tumor blood vessels [28]. However drugs with a long half-life in the circulation may establish a more uniform distribution in tissues even if penetration of tissue is relatively slow, whereas drugs with a short half-life may have a non-uniform distribution. Here we report a study of the distribution of the monoclonal antibodies, cetuximab and trastuzumab, in tumors that express different levels of their target receptors.

## Methods

### Drugs and reagents

The monoclonal antibody cetuximab (IMC-C225, Erbitux) was provided by Imclone Systems, Inc. (New York, NY, USA) as a solution at a concentration of 2 mg/ml. Trastuzumab (Herceptin) was obtained from the hospital pharmacy at a concentration of 21 mg/ml. The hypoxia-selective agent EF5 and Cy5-conjugated anti-EF5 antibody [29,30] were kindly provided by Dr. C. Koch, Philadelphia, PA. Blood vessels in tumor sections were visualized with a rat anti-mouse CD31 (PECAM-1) monoclonal antibody that was purchased from BD Pharmingen (Mississauga, ON, Canada) and the Cy3-conjugated goat anti-rat IgG secondary antibody was purchased from Jackson Immuno Research Laboratories, Inc. (West Grove, PA). Cetuximab and trastuzumab were recognized in tissue sections with goat anti-human IgG conjugated with horseradish peroxidase (Biosource, Montreal, Canada).

### Cell lines and tumor models

Experiments were performed utilizing the ErbB1-overexpressing human epidermoid carcinoma (A431) and a human breast adenocarcinoma (MDA-MB-231), using both wild-type and *ERBB2 *transfected (231-H2N) cell lines. A431 and MDA-MB-231 cells were obtained from the American Type Culture Collection (Manassas, VA, USA), while MDA-MB-231 cells transfected with *ERBB2 *(231-H2N) were kindly provided by Dr. J. Medin [31] (University of Toronto, ON, Canada). All the cell lines were maintained as monolayers in Dulbecco's Modified Eagle's Medium (DMEM), supplemented with 10% fetal calf serum (FCS), at 37°C in a humidified atmosphere of 95% air plus 5% CO_2_. Tests were performed routinely to ensure that cells were free of mycoplasma. Tumors were generated by injection of ~2 × 10^6 ^exponentially-growing cells into the right and left flanks of 6-8 week old female athymic nude mice, purchased from Harlan Sprague-Dawley Laboratory Animal Centre (Madison, WI, USA). Mice were housed five per cage, and sterile tap water and food were given ad libitum. All procedures were carried out following approval of the Institutional Animal Care Committee.

Expression of ErbB1 and ErbB2 receptors in the xenografts was confirmed by applying cetuximab or trastuzumab to sections of tumors ex vivo, followed by their recognition using anti-human IgG as described below. Endogenous expression of ErbB1 and ErbB2 were also confirmed and assessed by diagnostic antibodies from Zymed (Clone 31G7) and Neomarkers (Clone SP3) respectively.

### **Experimental design**

Tumor-bearing mice were divided randomly into groups of 5-6, and treatment with cetuximab or trastuzumab was initiated when the diameter of tumors was approximately 7-8 mm. One group was selected randomly as the control, and the other mice received cetuximab or trastuzumab (0.01 mg to 1.0 mg) as a single intraperitoneal (i.p.) or intravenous (i.v.) injection. Control mice were given equal volumes of PBS. Animals were killed at various intervals after injection of cetuximab or trastuzumab; they received an i.p. injection of EF5 (0.2 ml of 10 mM EF5) 2 hours before they were killed in order to identify hypoxic regions of tumors [29,30]. Tumors were removed and embedded with Tissue-Tek OCT (Optimal Cutting Temperature, Sakura Finetek USA Inc., Torrance, CA). The tissue boxes were gently immersed in liquid nitrogen, and then stored at -70°C.

Cryosections were prepared at 10 μm thickness and triple stained to identify cetuximab or trastuzumab, CD31 and EF5. Horseradish peroxidase (HRP) conjugated to anti-human IgG was used to recognize the therapeutic monoclonal antibodies. DAB (3,3'-diaminobenzidine) is a chromogenic substrate for HRP and it deposits a brown specific stain in the presence of HRP. Blood vessels in tissue sections were recognized by the expression of CD31 on endothelial cells. Purified rat anti-mouse CD31 monoclonal antibody was applied at a concentration of 1:500 and left overnight at 4°C. Primary antibody binding was disclosed using a Cy3-conjugated goat anti-rat IgG secondary antibody. Hypoxic regions were recognized by cyanine-5-conjugated mouse anti-EF5 (1/50) antibody.

### Fluorescence microscopy

Images were tiled using an Olympus BX50 upright fluorescent microscope linked to a Photometrics CoolSnap HQ2 CCD camera, a motorized X-Y stage connected to a computer preloaded with Media Cybernetics In Vivo and Image Pro-PLUS software (Media Cybernetics, Silver Spring, MD) and a stage controller board. Tumor sections were scanned and tiled under white light and two different filters: (i) images of Cy3 fluorescence of CD31 were visualized using 530 nm to 560 nm excitation and 573 nm to 647 nm emission filter sets, while (ii) images of the Cy5 fluorescence of EF5 were visualized with 630 nm to 650 nm excitation and 665 nm to 695 nm emission filter sets. Composite images of cetuximab, CD31, and EF5 or trastuzumab, CD31 and EF5 were generated using Image Pro PLUS (version 5) and subsequently pseudo-colored. To investigate the distribution of drug in relation to distance from the nearest blood vessel or hypoxic region, images displaying anti-CD31 staining or EF5 staining were converted to black and white binary images: each image was overlayed with the corresponding field of view displaying drug intensity, resulting in an 8-bit black and white image with blood vessels or hypoxic regions identified by an intensity of 255 (white) and drug intensity ranging from 0-254 (gray scale). Areas of interest were selected from each tissue section and were on average 1600 × 1600 μm (0.4 μm^2^/pixel). Areas of necrosis and staining artifact were excluded.

Distributions of each monoclonal antibody in relation to distance from the nearest blood vessel and the nearest region of hypoxia in the tumor section were quantified utilizing Image Pro software. A minimum signal level just below threshold was set for each tissue section; this was based on an average background reading from regions without staining. The pixel intensity and distance to the nearest vessel or region of hypoxia for all pixels within the selected region of interest above threshold were measured with a customized algorithm. The intensity of cetuximab or trastuzumab signal was represented as mean ± SEM for all pixels at a given distance to the nearest vessel or region of hypoxia and plotted as a function of that distance.

## Results

### Expression of ErbB receptors

**Figure 1:**
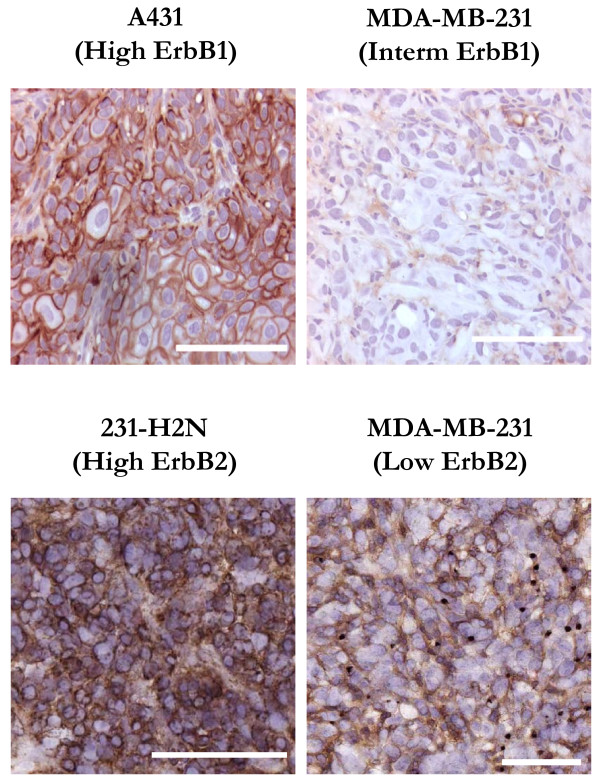
**Immmunohistochemical staining of sections of xenografts**. Immmunohistochemical staining after ex vivo application of cetuximab to identify ErbB1 expression (upper panels) or of trastuzumab to identify ErbB2 expression (lower panels). *Scale bar *= 100 μm.

Ex vivo staining using cetuximab was used to recognize expression of ErbB1 in A431 and MDA-MB-231 tumor sections; these tumors express high and intermediate levels of ErbB1 respectively (Fig. 1, upper panels). Similarly, ex vivo application of trastuzumab indicates low expression of ErbB2 in wild-type MDA-MB-231 xenografts and high expression in the *ERBB2*-transfected 231-H2N xenografts (Fig. 1, lower panels). In tumors that express the receptors the staining indicates their distribution on the cell membrane.

Expression of receptors was fairly uniform in tumors, except for regions of hypoxia (defined by EF5 staining) where there was lower expression of ErbB1 and ErbB2. We also studied receptor expression in tumors of animals that were treated with the therapeutic antibodies, and found no effect of treatment on receptor expression.

### Time- and dose-dependent distribution of cetuximab

**Figure 2:**
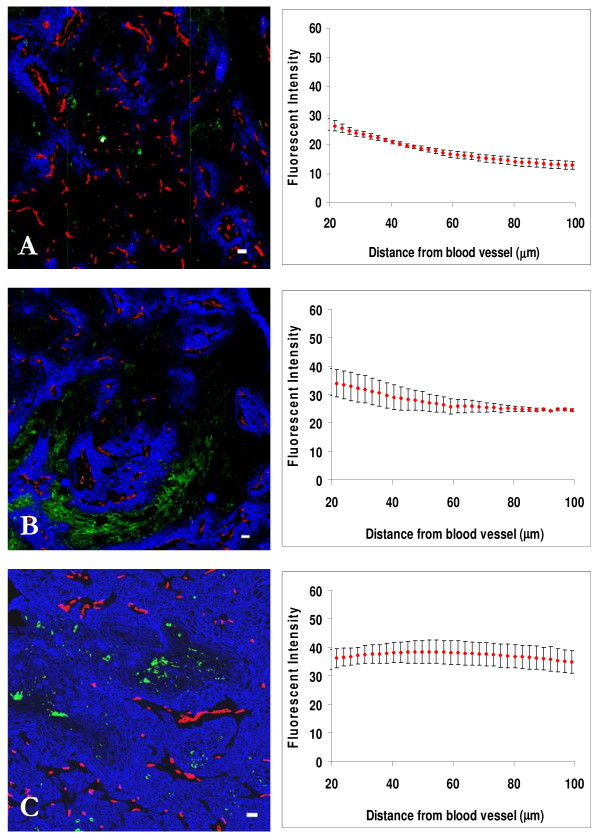
**Dose response distribution of cetuximab in relation to blood vessels and regions of hypoxia in A431 xenografts**. Left panels show the distribution of cetuximab (blue) in relation to blood vessels (red) and regions of hypoxia (green) in A431 xenografts at 24 h after an i.p. injection of (A) 0.01 mg, (B) 0.05 mg, and (C) 1.0 mg. In right panels staining intensity (mean +/- SEM) due to cetuximab is plotted against distance from the nearest blood vessel in the tumor section. Note minimal drug binding in hypoxic regions. *Scale bar *= 100 μm.

Dose-dependent distribution of cetuximab in A431 xenografts 24 h after i.p. injection of different doses is shown in Fig. 2. After injection of 0.01 mg or 0.05 mg cetuximab, there was selective distribution closer to blood vessels, and no penetration to hypoxic regions (shown in green), but at 24 h after injection of 1.0 mg cetuximab, the distribution was more uniform within the tumor, although there remained minimal drug penetration to hypoxic regions identified by uptake of EF5. Staining was honeycomb in appearance, consistent with antibody binding to receptors on the outer membranes of tumor cells. There was an apparent increase in intensity at very short distances from the centers of blood vessels, likely because of lack of expression of ErbB1 on endothelial cells and pericytes.

**Figure 3:**
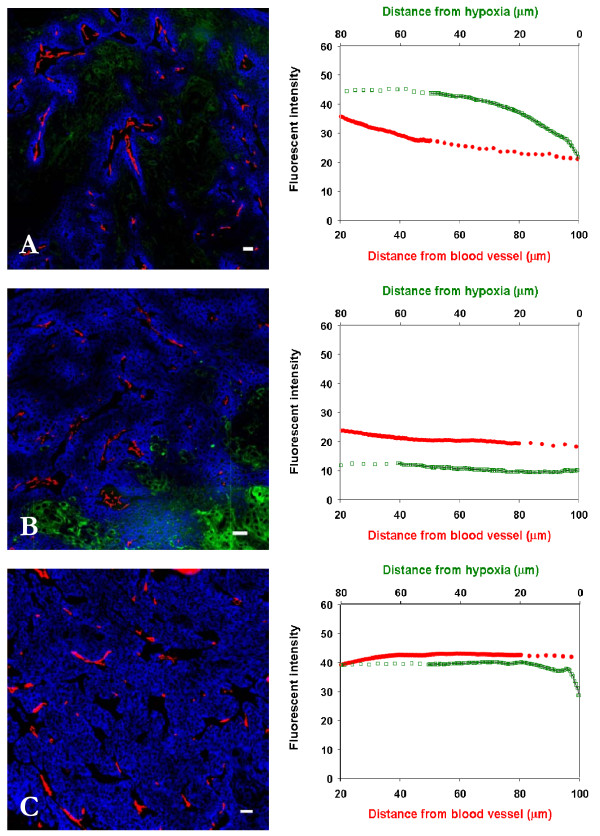
**Time response distribution of cetuximab in relation to blood vessels and regions of hypoxia in A431 xenografts**. Left panels show the distribution of cetuximab (blue) in relation to blood vessels (red) and regions of hypoxia (green) in A431 xenografts at (A) 30 min, (B) 4 h and (C) 48 h after i.p. injection of 1.0 mg. In right panels staining intensity due to cetuximab is plotted against distance to the blood vessel in red and distance to region of hypoxia in green. *Scale bar *= 100 μm.

The time-dependent distribution of cetuximab after an i.p. injection of 1.0 mg into mice bearing A431 xenografts is shown in Fig 3. With exclusion of the immediate perivascular region, there was a gradient of decreasing concentration at increasing distances from blood vessels at 30 min and 4 h after injection (Fig 3), but at 24 h and 48 h the intensity of cetuximab staining was relatively uniform within the tumor tissue (Figs. 2 and 3). There was no staining due to cetuximab in hypoxic regions shown in green. Cetuximab distribution in relation to hypoxic regions is also plotted in Fig 3, which shows that staining intensity due to cetuximab increases as the distance from the hypoxic regions increases. The slopes of the relationships between staining intensity of cetuximab and distance from blood vessels after various doses and times are summarized in Table 1. These data confirm relatively uniform distribution at 24 h - 48 h after injection of the higher dose of 1 mg cetuximab, with the caveat that there is still minimal binding within hypoxic regions of the tumors.

**Table 1:** Cetuximab and trastuzumab staining intensity in different xenografts.

Cell line	Monoclonal antibody	Dose (mg)	Time after injection	Staining Intensity at 20 μm from blood vessels (mean IU) ± SEM	Staining Intensity at 100 μm from blood vessels (mean IU) ± SEM	Gradient of Staining Intensity (IU/μm)
A431	Cetuximab	0.01	24 h	26.8 ± 2.0	12.7 ± 1.4	-0.18
		0.05	24 h	34.3 ± 4.8	24.5 ± 0.4	-0.12
		1.0	30 min	36.1 ± 0.2	21.2 ± 1.3	-0.19
		1.0	4 h	34.9 ± 2.7	25.0 ± 2.3	-0.12
		1.0	24 h	35.9 ± 3.5	34.7 ± 3.9	-0.02
		1.0	48 h	36.1 ± 1.2	37.8 ± 1.8	-0.02

MDA-MB-231	Cetuximab	0.01	24 h	7.0 ± 0.6	7.4 ± 1.1	0.01
		0.05	24 h	7.6 ± 0.8	6.9 ± 0.9	-0.01
		0.1	24 h	15.0 ± 2.8	18.5 ± 2.8	0.04
		0.5	15 min	6.9 ± 1.5	6.3 ± 0.7	-0.01
		0.5	30 min	8.3 ± 5.7	5.7 ± 3.7	-0.03
		0.5	1 h	18.9 ± 1.1	17.0 ± 1.1	-0.02
		0.5	2 h	19.1 ± 3.9	14.0 ± 2.9	-0.06
		0.5	4 h	20.8 ± 1.5	24.3 ± 1.5	0.04
		0.5	6 h	17.6 ± 4.0	20.3 ± 4.6	0.03
		0.5	24 h	16.7 ± 2.2	20.7 ± 1.6	0.05
		1.0	24 h	17.3 ± 2.7	21.2 ± 2.5	0.05

231-H2N	Trastuzumab	0.1	2 h	16.8 ± 2.1	12.7 ± 1.8	-0.05
		0.3	30 min	19.4 ± 0.9	16.6 ± 1.3	-0.04
		0.3	2 h	23.0 ± 1.6	19.9 ± 2.1	-0.04
		0.3	4 h	24.0 ± 2.1	18.5 ± 3.0	-0.07
		0.3	24 h	29.0 ± 1.0	27.3 ± 0.8	-0.02
		1.0	2 h	27.0 ± 1.3	26.2 ± 1.4	-0.01

Staining intensity of cetuximab and trastuzumab at 20 μm and 100 μm from blood vessels in A431, MDA-MB-231 and 231-H2N xenografts. Gradient of staining intensity is shown.

The distribution of cetuximab 24 h after an intravenous injection of different doses was also investigated in A431 xenografts (data not shown). There were no significant differences in the distributions of cetuximab after i.p. and i.v. injection.

Time- and dose-dependent distribution of cetuximab in MDA-MB-231 xenografts (which express intermediate levels of ErbB1) is summarized in Table 1: with exclusion of the immediate perivascular region, staining intensity was relatively constant with increasing distance from the blood vessel at most times and doses, suggesting more rapid distribution than in the A431 tumors, which have higher levels of expression of ErbB1. Absolute levels of bound cetuximab increased with both dose injected and time after injection.

### Time- and dose-dependent distribution of trastuzumab

**Figure 4:**
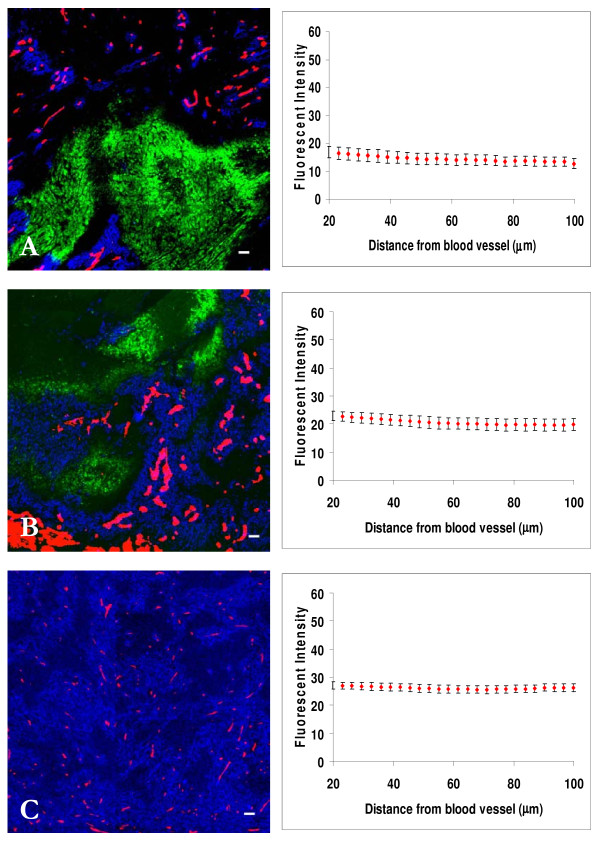
**Dose response distribution of trastuzumab in relation to blood vessels and regions of hypoxia in 231-H2N xenografts**. Left panels show the distribution of trastuzumab (blue) in relation to blood vessels (red) and regions of hypoxia (green) in MDA-MB-231 breast cancer xenografts transfected with ErbB2 (231-H2N) at 2 h after i.v. injection of (A) 0.1 mg, (B) 0.3 mg and (C) 1.0 mg. In right panels staining intensity (mean +/- SEM) due to trastuzumab is plotted against distance from the nearest blood vessel in the tumor section. Note minimal drug binding in hypoxic regions. *Scale bar *= 100 μm.

The distribution of trastuzumab at 2 h after i.v. injection of doses of 0.1 mg, 0.3 mg or 1.0 mg into mice bearing 231-H2N xenografts (which over-express ErbB2) is shown in Fig 4. There was selective localization close to blood vessels at lower doses and uniform distribution after the 1.0 mg dose. Staining due to trastuzumab was not found in regions of hypoxia (shown in green). Staining intensities at ~20 μm from blood vessels varied by a factor of ~1.5 after i.v. injection of doses of 0.1 mg - 1.0 mg (Table 1), suggesting that binding to proximal cells is close to saturated.

**Figure 5:**
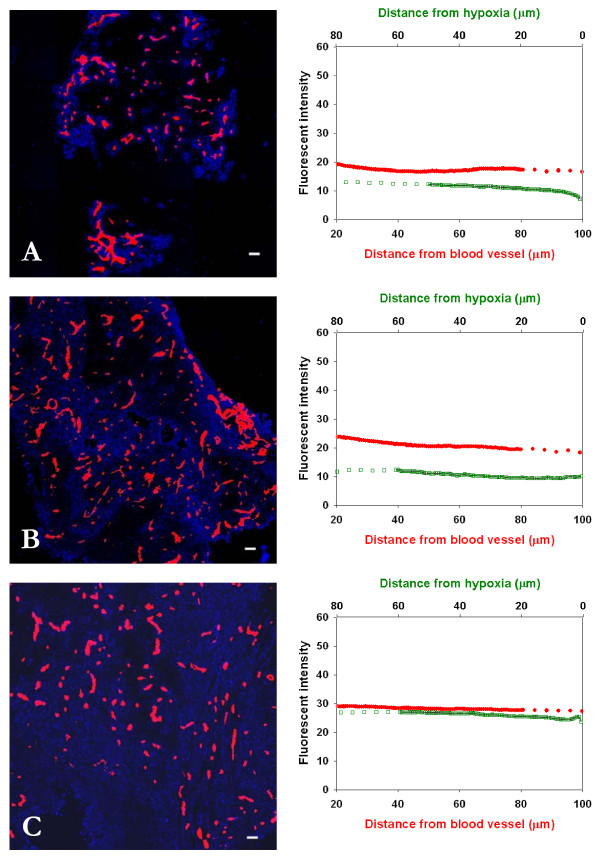
**Time response distribution of trastuzumab in relation to blood vessels and regions of hypoxia in 231-H2N xenografts**. Left panels show the distribution of trastuzumab (blue) in relation to blood vessels (red) in MDA-MB-231 breast cancer xenografts transfected with ErbB2 (231-H2N) at (A) 30 min, (B) 4 h and (C) 24 h after i.v. injection of 0.3 mg trastuzumab. In right panels staining intensity due to trastuzumab is plotted against distance to the blood vessel in red and distance to region of hypoxia in green. *Scale bar *= 100 μm.

The distribution of trastuzumab as a function of time after injection of 0.3 mg is shown in Fig 5: There was selective perivascular localization of trastuzumab at 30 min and 4 h after injection, but more uniform distribution after 24 h. Staining intensities at ~20 μm from blood vessels after an injection of 0.3 mg of trastuzumab varied only by a factor of ~1.5 at 30 min to 24 h after injection, again suggesting early saturation of cells proximal to blood vessels. Trastuzumab distribution in relation to hypoxic regions is plotted in green in Fig 5, staining intensity due to trastuzumab increases in regions close to hypoxia as the time interval increases.

Trastuzumab was not found bound to cells of MDA-MB-231 xenografts which express low levels of ErbB2.

## Discussion

Cetuximab and trastuzumab have shown limited efficacy in causing remission in a proportion of patients with metastatic colorectal cancer and breast cancer respectively [21,22], while trastuzumab has improved survival of women with ErbB2 positive breast cancer when given as adjuvant therapy after chemotherapy [32-34]. Monoclonal antibodies are large molecules, which are "consumed" by binding to receptors on the cell surface, conditions that might lead to poor penetration of tissue within solid tumors [28]. Indeed, an early study of the distribution of a radiolabeled monoclonal antibody into multicellular spheroids suggested very slow penetration of tissue, with establishment of a steep concentration gradient [35], and more recent studies of the penetration of drugs such as doxorubicin (which binds avidly to DNA) have shown quite poor distribution [23-25]. Thus limited distribution of therapeutic agents within solid tumors is a potentially important and relatively neglected cause of drug resistance, especially in the metastatic setting. Here we have used quantitative immunohistochemistry to study the distribution within human tumor xenografts of two therapeutic monoclonal antibodies in clinical use, cetuximab and trastuzumab, to determine if their efficacy might be limited by failure to reach all of the target tumor cells in an effective concentration.

The results of our study show that distribution of both of these therapeutic antibodies is time and dose-dependent. At short intervals after injection of all doses there is a concentration gradient of staining intensity of the antibodies with increasing distance from blood vessels within tumors that strongly express the target receptor. However there is a greater change in the gradient of cetuximab intensity in A431 xenografts than of trastuzumab intensity in 231-H2N xenografts. At moderate and high doses the distribution then becomes more uniform with time, while at lower doses the heterogeneous distribution is retained. Distribution of cetuximab and trastuzumab in relation to hypoxic regions provides a better understanding of the distribution of the antibodies distal to blood vessels. There remains minimal drug distribution to hypoxic tumor cells under all conditions, which is probably due both to limited availability of drug in these regions, and to decreased expression of the ErbB receptors under hypoxic conditions.

The difference in time dependence of the distributions of the monoclonal antibodies as compared to that for doxorubicin, which is relatively independent of time after injection [24] is most likely due to the half-lives of the drugs in the circulation: doxorubicin has a short initial half-life [36], such that most penetration from vessels takes place quickly, whereas monoclonal antibodies have a half-life of days [37-39], allowing for a more constant process of tissue penetration.

The gradients of cetuximab intensity in MDA-MB-231 xenografts, which express intermediate levels of ErbB1, are less steep than in A431 xenografts, which express higher levels of ErbB1, and homogeneity of distribution of cetuximab in MDA-MB-231 xenografts was achieved more rapidly. This is probably due to the low receptor binding of cetuximab (i.e. less consumption of drug) by proximal cells in MDA-MB-231 xenografts. Trastuzumab was not identified after injection in MDA-MB-231 xenografts, which express low levels of ErbB2.

Multiple phase I and II clinical trials have established that standard weekly dosing of cetuximab or trastuzumab in humans achieves trough serum concentrations that are usually above 50 μg/ml [37,38,40-42]. We did not measure serum concentration of cetuximab or trastuzumab in our mice. Others have reported maximum serum levels of cetuximab of ~65 μg/ml and ~400 μg/ml cetuximab after injection of doses of 0.25 mg and 1.0 mg into mice respectively [28,39], similar to those reported in patients. Injection of trastuzumab was reported to lead to serum levels of about 5 ng/ml at 6-24 hours after i.p injection of a single low dose of 0.3 mg/kg into mice [43]; if pharmacokinetics were linear this would imply doses of ~15 mg/mouse to achieve levels of 10 ug/ml in serum, but it seems unlikely that pharmacokinetics of the two antibodies would differ by such a large amount.

Several other investigators have studied the distribution of various antibodies, or antibody fragments, in tumors. Their results depend on changes in blood flow [44] the affinity of the antibodies for their targets, but in general these authors have reported problems of heterogeneity of distribution at various times after their administration [45-48]. We were able to identify two other studies of the distribution of trastuzumab (but none of cetuximab) in solid tumors. Dennis et al used intravital microscopy to detect trastuzumab, conjugated to fluorescein isothiocyanate (FITC), in relation to blood vessels of MMTV/HER2 transgenic mice (expressing high levels of ErbB2) that were constrained to grow in a transparent window chamber; they reported perivascular localization of trastuzumab at 24 hours after injection of 10 mg/kg (about 0.25 mg/mouse) [49]. Their study suggests poorer (or slower) distribution of trastuzumab than the one reported here; a possible reason is higher expression of ErbB2 in the MMTV/HER tumors as compared to the 231-H2N xenografts investigated in our study. Baker et al used similar methods to our own, and investigated time-dependent distributions of trastuzumab in xenografts (that did or did not express ErbB2) after i.p. injection doses in the range of 4-20 mg/kg (about 0.1- 0.5 mg/mouse) [50]. They found perivascular distribution of drug at 3 h, and that tumor margins reached saturation with trastuzumab more rapidly than the (poorly-vascularized) interior. Drug distribution became more uniform at 24 h as compared to 8 h after injection of 4 mg/kg, but some heterogeneity of trastuzumab distribution was observed in the tumor under all conditions; this is consistent with our finding of poor drug uptake in hypoxic tumor regions.

## Conclusions

Limited distribution of anticancer drugs (including molecular targeted agents) to cells within human tumors is an important mechanism that may lead to clinical drug resistance. The present study suggests that while distribution of cetuximab and trastuzumab within tumor tissue is time and dose-dependent, the sustained concentrations achieved by repeated dosing in patients is likely to achieve relatively uniform concentration within most areas of tumors, although there is poor drug binding in hypoxic regions. Thus the presence of hypoxia may be associated with resistance to these targeted agents, as well as to radiotherapy and chemotherapy.

## Competing interests

The authors declare that they have no competing interests.

## Authors' contributions

CL designed and performed all the experiments and drafted the manuscript. IT conceived of the study, obtained funding for it and participated in its design and coordination and drafted the manuscript. Both authors read and approved the final manuscript.

## Pre-publication history

The pre-publication history for this paper can be accessed here:

http://www.biomedcentral.com/1471-2407/10/255/prepub
